# E6/E7 mRNA Expression of the Most Prevalent High-Risk HPV Genotypes in Cervical Samples from Serbian Women

**DOI:** 10.3390/diagnostics13050917

**Published:** 2023-02-28

**Authors:** Natasa Nikolic, Branka Basica, Aljosa Mandic, Nela Surla, Vera Gusman, Deana Medic, Tamas Petrovic, Mirjana Strbac, Vladimir Petrovic

**Affiliations:** 1 Institute of Public Health of Vojvodina, 21000 Novi Sad, Serbia; 2 Clinic for Oncological Surgery, Oncology Institute of Vojvodina, 21208 Sremska Kamenica, Serbia; 3 Department of Gynaecology and Obstetrics, Faculty of Medicine, University of Novi Sad, 21000 Novi Sad, Serbia; 4 Department of Microbiology with Parasitology and Immunology, Faculty of Medicine, University of Novi Sad, 21000 Novi Sad, Serbia; 5 Scientific Veterinary Institute Novi Sad, 21000 Novi Sad, Serbia; 6 Department of Epidemiology, Faculty of Medicine, University of Novi Sad, 21000 Novi Sad, Serbia

**Keywords:** E6, E7, HPV, cervical intraepithelial lesion, biomarker, Serbia

## Abstract

Cervical cancer caused by persistent infection with HR HPV genotypes is the second leading cause of death in women aged 15 to 44 in Serbia. The expression of the E6 and E7 HPV oncogenes is considered as a promising biomarker in diagnosing high-grade squamous intraepithelial lesions (HSIL). This study aimed to evaluate HPV mRNA and DNA tests, compare the results according to the severity of the lesions, and assess the predictive potential for the diagnosis of HSIL. Cervical specimens were obtained at the Department of Gynecology, Community Health Centre Novi Sad, Serbia, and the Oncology Institute of Vojvodina, Serbia, during 2017–2021. The 365 samples were collected using the ThinPrep Pap test. The cytology slides were evaluated according to the Bethesda 2014 System. Using a real-time PCR test, HPV DNA was detected and genotyped, while the RT-PCR proved the presence of E6 and E7 mRNA. The most common genotypes in Serbian women are HPV 16, 31, 33, and 51. Oncogenic activity was demonstrated in 67% of HPV-positive women. A comparison of the HPV DNA and mRNA tests to assess the progression of cervical intraepithelial lesions indicated that higher specificity (89.1%) and positive predictive value (69.8–78.7%) were expressed by the E6/E7 mRNA test, while higher sensitivity was recorded when using the HPV DNA test (67.6–88%). The results determine the higher probability of detecting HPV infection by 7% provided by the mRNA test. The detected E6/E7 mRNA HR HPVs have a predictive potential in assessing the diagnosis of HSIL. The oncogenic activity of HPV 16 and age were the risk factors with the strongest predictive values for the development of HSIL.

## 1. Introduction

It is estimated that approximately every fourth malignancy can be linked to an infectious agent, that is, its contribution to various stages of cancer development (reviewed in [1]). About a third of this contribution is related to the human papillomavirus (HPV) (reviewed in [2]). Today, significant evidence confirms the association of high-risk (HR) HPV as a carcinogen or promoter in developing malignant diseases in different locations: the cervix, vulva, vagina, penis, anus, and certain head and neck regions. In first place are neoplasias of the lower genital tract, such as cervical cancer [3]. According to estimates by the World Health Organization (WHO), that is, by the International Agency for Research on Cancer (IARC), 604,000 new cases and 342,000 deaths were registered around the world in 2020, which makes cervical cancer the fourth most frequently diagnosed cancer in women [4]. In Serbia, organized cervical cancer screening has been conducted since 2012, using the PAP test, based on the cytomorphological examination of cervical samples. Screening is mandatory for women aged 25 to 69. However, despite organized screening, cervical cancer remains one of the most common cancers among women in Serbia [5]. The incidence of cervical cancer in Serbia is still among the highest and is approximately twice the average in Europe (10.7 to 100,000) [6,7]. It is necessary to emphasize that data on the HPV prevalence and genotype distribution among women with normal cervical cytology, precancerous cervical lesions, and cervical cancer are missing in the updated IARC Human Papillomavirus and Related Diseases Report for Serbia [6].

HPV vaccination is a crucial prevention tool against HPV infection and HPV-related precancers and cancers [8]. If vaccination against HPV is carried out before initial sexual activities, it is one of the most effective ways to prevent cervical cancer [9]. Still, in Serbia, vaccination against HPV infection is not part of the mandatory national immunization program, but it is recommended for children aged 9 to 19 years [5].

Strong evidence for HPV as a causative aetiology of cancers of various locations was provided by the IARC, which classified HPV according to its potential to cause malignant cell alteration as follows [10]:

Group 1 (carcinogenic to humans, HR) includes HPV genotypes: 16, 18, 31, 33, 35, 39, 45, 51, 52, 56, 58, and 59;

Group 2A (probably carcinogenic) includes HPV genotype 68;

Group 2B (potentially carcinogenic) includes HPV genotypes: 26, 53, 66, 67, 70, 73, 82, 30, 34, 69, 85 and 97;

Group 3 (low risk, LR) includes HPV genotypes 6 and 11.

Persistent HPV infection is the most critical risk factor for the development of cervical cancer, which is confirmed by the presence of HR HPV in over 99% of cervical cancer samples. Concerning the oncogenic potential, infection with a particular HR HPV genotype carries a specific risk for cellular transformation and malignancy (reviewed in [3]). Namely, one of the most critical determinants of the degree of pathogenicity of different HPV genotypes is the functional differences between their oncoproteins, E6 and E7 [11].

During viral genome integration into the host cell genome, E1 or E2 are usually disrupted [12]. This gene disruption leads to uncontrolled transcription of E6 and E7 genes as the E2 repression on these oncogenes disappears [13,14]. Their protein products lead to unregulated cell proliferation, differentiation, and loss of the reparative abilities of the host cell, wherefore they are considered the main actors of virus-induced oncogenesis of cervical cancer [15,16]. Thanks to the use of cervical cancer screening tests, this cancer is classified as one of the most preventable malignancies. The most common test for this purpose is the cytological abnormality test, the Papanicolaou (PAP) test. However, considering the etiological role of HR HPV in developing cervical cancer, DNA tests have been incorporated into the primary screening of developed countries. Still, this test is characterized by high sensitivity and low specificity, which indicates the necessity of improving the test’s characteristics concerning specificity [17]. In this context, the results of numerous studies state that using the HR HPV mRNA test as a basic or additional test in primary screening would improve these characteristics [18].

Given the above, this research aimed to determine the oncogenic activity of the most commonly diagnosed HR HPVs in cervical smear samples using the mRNA test and compare the results according to the severity of the cervical intraepithelial lesion. Furthermore, it aimed to examine the clinical characteristics and predictive potential in assessing the diagnosis of high-grade cervical intraepithelial lesions of HPV DNA and mRNA tests.

## 2. Materials and Methods

### 2.1. Study Population and Specimen Collection

From 2017 to 2021, cervical smears were obtained from a sample of 365 female patients (age 20–74 years) with normal and abnormal results of cervical cytology who were undergoing gynecological exams at the Department of Gynecology, Community Health Centre Novi Sad, Serbia, and the Oncology Institute of Vojvodina, Serbia. All of the women in the study did not receive any prior treatment for cervical dysplasia or cancer, and all were unvaccinated against HPV infection. The samples were collected using the ThinPrep Pap test (Hologic Inc.) according to the manufacturer’s instructions and sent for further analyses to the Center of Virology, Institute of Public Health of Vojvodina, Novi Sad, Republic of Serbia.

The classification of cytological findings was performed according to the criteria of the Bethesda System 2014. It was categorized into negative for intraepithelial lesion or malignancy (NILM), atypical squamous cells of unknown significance (ASCUS), low-grade squamous intraepithelial lesions (LSIL), and high-grade squamous intraepithelial lesions (HSIL). All of the women enrolled in the study were informed about the research objective and signed an informed written consent form. The study protocol was reviewed and approved by the Medical Ethical Committee of the Institute of Public Health of Vojvodina, Novi Sad, Serbia (approval number: 01-252/3).

### 2.2. HR HPV Detection and Genotyping

The ThinPrep cervical smear samples were stored at 4–8 °C for up to 3 days from the sampling day. The 2 mL of collected samples were transferred to nuclease-free tubes and centrifugated at 8000× *g* for 5 min. The formed pellet was dissolved in 200 µL of nuclease-free water and used for nucleic acid extraction. According to the manufacturer’s instructions, DNA extraction was carried out using the SaMag STD DNA Extraction Kit (Sacace Biotechnologies, Como, Italy). The extracted DNA was eluted in 100 μL elution buffer. The detection and genotyping of 12 HR HPV genotypes (16, 18, 31, 33, 35, 39, 45, 51, 52, 56, 58, and 59), marked as the HPV DNA test, were performed using the High Risk Typing Real-TM Kit (Sacace Biotechnologies, Como, Italy) following manufacturer’s instructions. The E7 gene of specific HPV genotypes was amplified using primers and TaqMan probes in the multiplex reaction performed in a total of 13 μL. The β globin gene is used as an internal control. Real-time PCR was performed on the SaCycler-96 (Sacace Biotechnologies, Como, Italy). After the initial activation of the DNA polymerase at 95 °C for 15 min, five cycles of amplification were performed under the following conditions: 95 °C/5 s, 60 °C/20 s, and 72 °C/15 s, and 40 amplifications were performed under the following conditions: 95 °C/5 s, 60 °C/30 s (fluorescence detection), and 72 °C/15 s. The kinetics of the detected fluorescence signals were monitored using the SaCycler-96 software package (Sacace Biotechnologies, Como, Italy).

### 2.3. E6/E7 mRNA HPV Detection

E6/E7 mRNA of the most prevalent HPVs was tested in the cervical samples positive for the most prevalent HR HPVs DNA and HR HPV DNA negative samples. The HR-HPV-negative samples were included in E6/E7 mRNA testing because the study aimed to determine the mRNA test’s clinical characteristics by evaluating and comparing it with the HPV DNA test. Total RNA was extracted from the prepared sample using the miRNeasy Mini Kit and QIAcube robotic workstation (Qiagen, Hilden, Germany) following the manufacturer’s instructions. The total RNA was eluted in 50 µL ultrapure water free from nucleases. Following the manufacturer’s recommendations, potentially present contaminants were removed using the TURBO DNA-free Kit (Invitrogen/ThermoFisher Scientific, Waltham, MA, USA). The routine procedure for removing contaminants using the kit above included the addition of 5 µL of 10× TURBO DNase Buffer and 1 µL of TURBO DNase enzyme into each sample of extracted total RNA, with incubation for 30 min at a temperature of 37 °C. After the action of the enzyme, 5 µL of inactivation reagent (Dnase Inactivation Reagent) was added, with incubation for 5 min, at room temperature and occasional vortexing. After that, centrifugation was performed (90 s, 10,000× *g*). The supernatant was carefully transferred to a nuclease-free tube. The real-time reverse transcription PCR (RT-PCR) analysis, marked as the HPV mRNA test, was performed using specific primers and TaqMan probes to detect the E6/E7 mRNA of individual HPV genotypes. The sequences for the primers and probes (Table 1) were adopted from Lindh et al. (2007) [19] and purchased from Life Technologies (Carlsbad, CA, USA). The AgPath-ID One-Step RT-PCR Kit (Applied Biosystems, Waltham, MA, USA) was used for the real-time RT-PCR. A separate reaction mixture was prepared for each set of primers and TaqMan probes. The reaction was prepared to a final volume of 25 μL containing: 12.5 μL 2× RT-PCR Buffer, 1 µL of 25× RT-PCR Enzyme Mix, primers to a final concentration of 300 nM, the probe to a final concentration of 200 nM, 1 µL of RNase Inhibitor reagent (Applied Biosystems, Waltham, MA, USA), 5 μL of isolated total RNA, and DEPC-treated nuclease-free water (Invitrogen, Waltham, MA, USA). Real-time PCR was performed on the Applied Biosystems 7500 Real-Time PCR Systems (ThermoFisher Scientific, Waltham, MA, USA). After the reverse transcription reaction at 48 °C for 30 min, the inactivation of reverse transcriptase and the activation of Taq polymerase were performed at 95 °C for 10 min. After that, 45 cycles of PCR amplification were carried out with denaturation at 95 °C for 15 s and annealing and elongation at 58 °C for 1 min. The data were analyzed with the Applied Biosystems Software v2.0.6 (ThermoFisher Scientific, Waltham, MA, USA) and the GraphPad Prism 8 (GraphPad Software, San Diego, CA, USA).

### 2.4. Statistical Analysis

All of the statistical analyses were performed using SPSS statistics software Version 21.0 (Chicago, IL, USA). Testing the difference in frequencies of attributive features was performed using the Chi-square (χ^2^) test of independence and quality of the match. The Student’s t-test was used to compare values between the two age groups, a numerical characteristic. A one-way analysis of variance (ANOVA) and the Bonferroni post-hoc test were applied to compare values between three or more data groups. Frequencies were used to present the analysis of the oncogenic activity of multiple HR HPV infections. Sensitivity, specificity, positive predictive value (PPV), negative predictive value (NPV), and their 95% confidence intervals (CIs) of HR HPVs DNA and E6/E7 mRNA HPVs detection and cytology test were calculated. To quantify the diagnostic capabilities of the selected test and evaluate its significance, the receiver operating characteristics (ROC) curve was used, which enables testing the significance of differences in the discriminating potential of different variables for the same binary outcome. It is based on a graphical presentation of pairs of sensitivity and specificity that can be obtained by estimating the threshold value for all values of discontinuous variables of the sample. Univariate and multivariate logistic regression were used to analyze the connection between two or more features, generating adequate statistical models. Multivariate logistic regression analysis was applied to all of the analyzed factors to construct a predictive model and named the most relevant predictors for the development of HSIL. A *p*-value of less than 0.05 defined as statistically significance.

## 3. Results

### 3.1. Cervical Cytology

A total of 365 specimens obtained from women in the north part of the Republic of Serbia (Vojvodina) were classified based on the Bethesda System 2014 into four categories by cytological criteria.

### 3.2. HR HPV DNA in Cervical Samples

**Figure 1:**
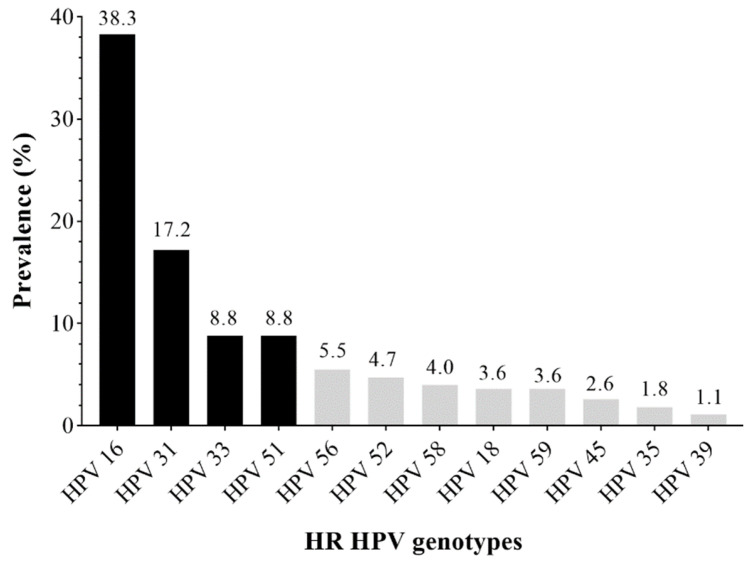
Genotype-specific distribution of HR HPVs.

The cervical samples were analyzed for 12 HR HPVs, where 246 out of 365 (67.4%) had HPV-DNA-positive results, which indicates that the overall prevalence of HPV in the study population was 67.4%. All of the HPV genotypes covered by the HPV DNA test were identified (*n* = 274) in the study population (246 HR-HPV-positive cervical samples). The most prevalent HPV genotype is HPV 16 which makes up 38.3% (105/274) of the total HPV-detected genotypes in 42.7% (105/246) of HP- DNA-positive samples. HPV 31 takes second place with 17.2% (47/274) of total HPV-detected genotypes in 19.1% (47/246) of HPV-DNA-positive samples. Equally represented are HPV 33 and HPV 51, each with 8.8% (24/274) of total HPV-detected genotypes in 9.8% (24/246) of HPV-DNA-positive samples (Figure 1). The results show that those HR HPVs make up 73% (200/274) of the detected genotypes, including multiple infections, which determined those cervical samples (*n* = 172) for further examination of oncogenic activity, according to the study’s aim. Multiple HPV infections were found in 15.7%. The most common co-infections were those with HPV 16 and 31, found in 7.6% (13/172) of cases with multiple infections (Table 2).

Molecular data were compared with the cytological results and age categories of patients. The distribution of cytological groups was analyzed within the most prevalent HR-HPV-DNA-positive samples (172 cervical samples), including multiple infections. A minority of women, 16.9% (*n* = 29), had normal results, whereas 83.1% (*n* = 143) showed different abnormalities. A total of 26.7% (*n* = 46) of the examined women had ASCUS; in 25.6% (*n* = 44), LSILs were found, whereas HSILs were detected in 30.8% (*n* = 53). The mean age of the patients was 36.7 years. Among the specimens, the number of Serbian women who were ≤30 years, 31–44 years, and ≥45 years old accounted for 36.5% (*n* = 68), 36.0% (*n* = 62), and 24.4% (*n* = 42) of the samples, respectively (Table 3).

The distribution of the most frequently detected HPVs concerning cytology is shown in Table 4. The prevalence rates of HR HPV 16 ranged from 44.8% in the group of NILM cytology to 75.5% in the HSIL group. Contrarily, the prevalence of HR HPV 31 is similar in the groups of NILM (37.9%), ASCUS (34.8%), and LSIL (31.8%), while it is lower in the group of HSIL (11.3%). HPV genotypes 33 and 51 are present in all of the cytological groups in less than 21%. The statistically significant difference in the prevalence between the number of positive findings of HPV 16 (χ^2^ test; *p* = 0.035) and HPV 31 (χ^2^ test; *p* = 0.017) was determined, depending on the degree of severity of the cytological findings, which was not determined for HPV 33 and 51 (χ^2^ test; *p* = 0.706, *p* = 0.790, respectively) (Table 4).

**Figure 2:**
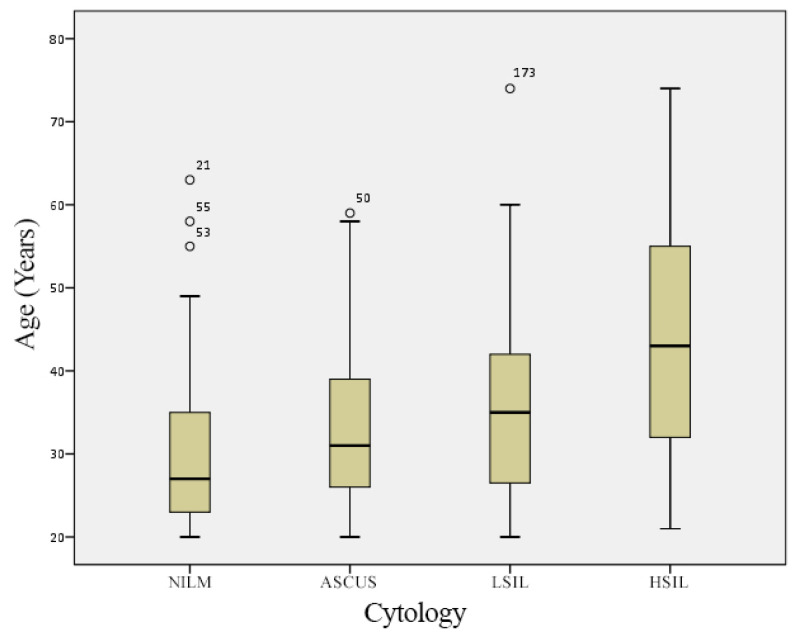
Age-specific analyses of the most prevalent HR HPV DNA in different cytological groups. NILM—negative for an intraepithelial lesion or malignancy; ASCUS—atypical squamous cells of unknown significance; LSIL—low-grade squamous intraepithelial lesions; HSIL—high-grade squamous intraepithelial lesions.

A statistically significant difference was found in the number of female patients concerning the cytological findings and the age of the patients (χ^2^ test; χ^2^ = 29.500; *p* = 0.000) (Table 5). The statistically significant difference was determined regarding the cytological findings and the age of the patients, where the female patients diagnosed with HSIL were significantly older compared to the other groups (ANOVA; F = 9.321; *p* < 0.001). The Bonferroni post-hoc test determined that the female patients with HSIL are statistically significantly older than those with ASCUS (*p* < 0.001), NILM (*p* < 0.001), and LSIL (*p* = 0.012) (Figure 2, Table 5). Female patients with confirmed HPV 31 are statistically significantly younger (33 years) than the other HR-HPV-DNA-positive patients (t = 2.317; *p* = 0.022). The average age of the female patients with confirmed HPV DNA 16 was 36.9 years; with HPV DNA 33, it was 34.1 years, while the average age of patients with HPV DNA 51 was 40.5 years. The statistical analyses show that the proportion of HPV 16 positivity maintained at the same level as age. Conversely, the proportion of HPV 31 positivity decreases with age. The detection of HR HPV 33 genotypes decreases with age, while HR HPV 51 increases (Table 5).

### 3.3. E6/E7 mRNA in Cervical Samples

**Figure 3:**
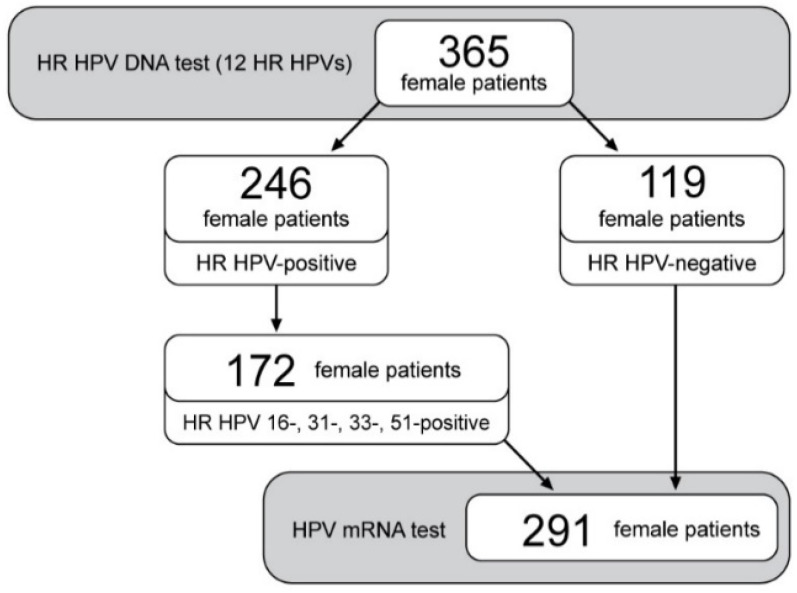
Flowchart presenting the study design. HR—high risk; HPV—human papillomavirus.

Figure 3 shows the study design with sample processing to analyze the expression of the E6/E7 mRNA of the most prevalent HR HPV in cervical samples from Serbian women. A total of 291 cervical samples, which include HPV 16-, 31-, 33- and 51-positive (*n* = 172) and HR-HPV-negative samples (*n* = 119), were tested by the HPV mRNA test. The E6/E7 mRNA HPV was detected exclusively in HR-HPV-DNA-positive samples (Table 6). E6 and E7 transcripts of the four most frequent HR HPVs were detected in 57.5% (115/200) of the HR HPV DNA confirmed genotypes. Accordingly, the distribution of E6/E7 mRNA HR HPV 16, 31, 33, and 51 are shown in Table 6. The E6/E7 mRNA HR HPV 16 was the most abundant, which accounted for 25.5% (51/200) of HR HPV genotypes. Next in frequency was the E6/E7 mRNA HR HPV 31 in 16.5% (33/200), while the E6/E7 mRNAs HR HPV 33 and 51 were equally represented in 8% (16/200) and 7.5% (15/200), respectively. Almost every second HPV 16 genotype is oncogenically expressed (48.6%; 51/105), and it was detected in 29.7% (51/172) HPV-DNA-positive samples. The oncogenic activity of HPV 31 was detected in approximately every fifth (19.2%; 33/172) HPV-DNA-positive sample. Regarding the oncogenic activity of the remaining tested genotypes, HPV 33 and HPV 51 were detected in roughly every tenth HPV-DNA-positive sample (Table 6). The results of expressing E6 and E7 HR HPV oncogenes were expressed through the dispersion of the obtained Ct values. The oncogenic activity of HPV 16 is detected by the lowest registered value (Ct = 16) (Supplementary Materials Figure S1).

### 3.4. Prevalence of HR HPV Based on E6/E7 mRNA HPV Expression in Different Cytological Groups

The expression of the E6 and E7 genes as indicators of the oncogenic activity of HR HPV 16, 31, 33, and 51 was analyzed concerning cytological findings. The oncogenic activity of the tested genotypes increases with the severity of the cervical intraepithelial lesion. A statistically significant difference in E6/E7 mRNA HPV expression among the various cytological groups was observed (χ^2^ test; χ^2^ = 108.623; *p* < 0.001). E6/E7 mRNA HPVs are the most prevalent in patients with HSIL cytological findings (88.9%). In the group of patients with LSIL cytological findings, it was demonstrated in a lower percentage (60%). A two-fold lower prevalence is observed in patients with ASCUS (29.4%). Oncogene activity in women with normal cytological findings is present in 10.9% of samples (Table 7). Subsequently, the E6/E7 mRNA distribution of HR-HPV-DNA-positive samples according to genotype and cytological groups was analyzed. E6/E7 mRNA HR HPV 16 is the most represented in patients with HSIL cytological findings (64.2%), while in the other groups of cytological findings, it was demonstrated in a lower percentage (3.4–22.7%). A statistically significant difference was found in the number of positive findings of E6/E7 mRNA HR HPV 16 concerning the cytological status (χ^2^ test; χ^2^ = 46.881; *p* < 0.001). This result singled out the HR HPV 16 genotype for further analyses. The presence of E6/E7 mRNA HR HPV 31 was the least detected in HSIL (7.5%) compared to other cytological groups (22.7–26.1%). The distribution of the oncogenic activity of the remaining genotypes (HR HPV 33 and 51) is approximately the same across different cytological groups and remains at a low level (2.2–11.4%) (Table 7).

The analyses of the oncogenic activity of multiple HR HPV infections are presented by frequencies. The overall oncogenic activity, including both single and multiple HR HPV infections detected using the E6/E7 mRNA HR HPV test, increases with the degree of cervical lesion severity (60–100%). The oncogenic activity detected in single-genotype infections (20.0–85.7%) is higher compared to the oncogenic activity of multiple genotypes (0–40%) (Table 8).

### 3.5. Prevalence of E6/E7 mRNA HR HPV Expression According to Age

The results were categorized according to age categories (≤30, 31–44, ≥45 years) to analyze the prevalence of E6/E7 mRNA HPV in the specific age groups. The lowest percent of E6/E7 mRNA HR HPV 16 was detected in the younger group and the highest percent was detected in patients over 44 years. A statistically significant difference was observed in patients with E6/E7 mRNA HR HPV 16 expression (χ^2^ test; χ^2^ = 7.331; *p* = 0.026), wherein individuals with positive E6/E7 mRNA HPV results were older than the others. The detection of E6/E7 mRNA HR HPV 31 and 33 decreases with the increasing age of the patient, while E6/E7 mRNA HR HPV 51 increases (Table 9).

### 3.6. Comparison of Tests for the Detection of HR HPV Genotypes and Their Oncogenic Activity

**Figure 4:**
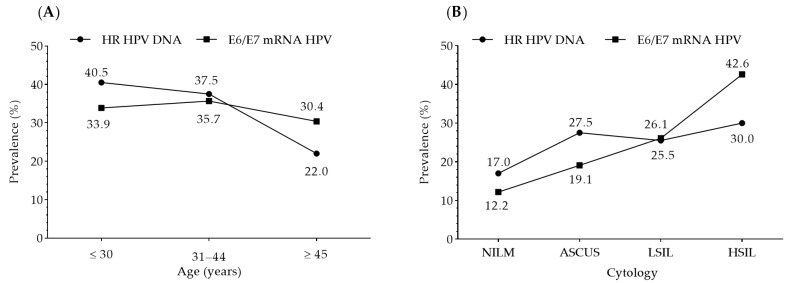
Prevalence of the most prevalent HR HPVs DNA and achieved oncogenic activity according to age (**A**) and cytology (**B**). NILM—negative for an intraepithelial lesion or malignancy; ASCUS—atypical squamous cells of unknown significance; LSIL—low-grade squamous intraepithelial lesions; HSIL—high-grade squamous intraepithelial lesions.

The comparison of tests for the detection of the most prevalent HR HPV genotypes and their oncogenic activity revealed that the presence of the HR HPV genotypes is higher than the presence of the oncogenic activity of the genotypes in younger women (≤30 years), similar in middle-aged women (31–44 years), and lower in women 45 years and older. The prevalence of oncogenic activity of the HPV genotypes increases with the severity of the cervical intraepithelial lesion. Compared to the prevalence of the examined HR HPV genotypes, the same parameter is lower in women with normal and undefined cytological findings, approximately the same in low-grade lesions, and significantly higher in high-grade lesions (Figure 4).

The calculated clinical characteristics of the DNA and E6/E7 mRNA HR HPV tests are shown in Table 10. The sensitivity and NPV of both tests were increased with the severity of the cervical intraepithelial lesion, with the HR HPV DNA test showing a statistically significantly higher level (67.6–98.2%). The specificity of the E6/E7 mRNA HR HPV test (89.1%) is statistically significantly higher than the HR HPV DNA test (75.6%). The PPV of the HR HPV DNA test is approximately the same for all types of cytological findings (60.3– 64.6%), while for the mRNA HR HPV test, it increases with the degree of the cervical intraepithelial lesion (69.8– 78.7%) and it is statistically significantly higher (Table 10).

**Figure 5:**
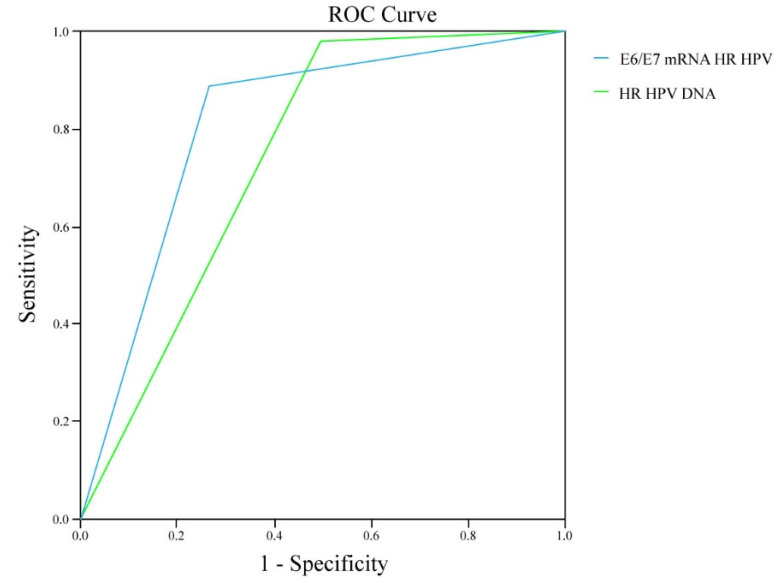
ROC curve of HR HPV and E6/E7 HR HPV tests in HSIL.

To quantify the diagnostic capabilities of the E6/E7 mRNA HPV test and evaluate its significance, an ROC curve was used to assess the assays for detecting HSIL. The area determined by the ROC curve (AUC) of E6/E7 mRNA HR HPV is 0.812 (CI (95%): 0.752–0.871), while the area under the ROC curve formed by the parameters of the HR HPV DNA test is 0.740 (CI (95%): 0.680–0.799) (Table 11, Figure 5).

**Figure 6:**
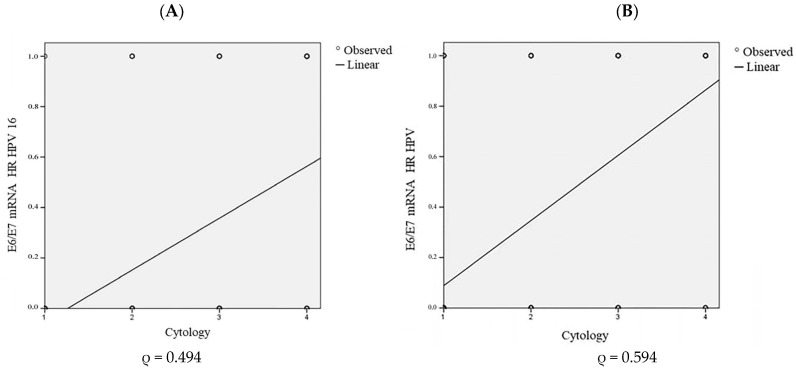
Correlation between the oncogenic activity of the HPV 16 genotype and cytology (**A**) and overall HPVs’ oncogenic activity and cytology (**B**). NILM (1), ASCUS (2), LSIL (3), HSIL (4), and Spearman’s correlation coefficient (ρ).

The relationships between the oncogenic activity of all of the tested HPVs and previously singled out HPV 16 vs. the cytological results were analyzed using Spearman’s (ρ) correlation (Figure 6). There is a statistically significant positive moderate correlation between the presence of HPV 16 oncogenic activity and cytological status (Spearman’s correlation; ρ = 0.494; *p* < 0.001) (Figure 6A). The oncogenic activity of the tested HPV genotypes is associated with a strong statistically significant positive correlation with the degree of cervical intraepithelial lesion severity (Spearman’s correlation; ρ = 0.594; *p* < 0.001) (Figure 6B).

Univariate multinomial logistic regression examined the individual factors (the HR HPV DNA 16, the oncogenic activity of all of the tested HR HPVs, the oncogenic activity of the HR HPV 16 genotype, and the age category) that indicated an increased probability of developing HSIL (Supplementary Materials Tables S1–S4).

For the influence of HR HPV 16 on the probability of HSIL, a statistically significant predictive value was determined in the NILM and LSIL cytological groups. Patients with the confirmed presence of the HPV 16 genotype through a DNA test have a higher probability of being diagnosed with HSIL than NILM (3.8-fold), while they will have a 2.6-fold higher probability of being diagnosed with HSIL compared to the probability of being diagnosed with LSIL (Supplementary Materials Table S1).

The influence of the oncogenic activity of all tested of the HR HPV genotypes (E6/E7 mRNA HR HPVs) on diagnosing high-grade lesions of the cervical epithelium has a statistically significant prediction found in all cytological groups, NILM, ASCUS, and LSIL. If patients have confirmed indicators of oncogenic activity, they will have an almost seven-fold higher probability of being diagnosed with HSIL compared to the probability of being diagnosed with NILM. The same patients have a 19-fold higher probability of being diagnosed with HSIL compared to the probability of detecting ASCUS and a 5-fold higher probability of detecting HSIL compared to LSIL (Supplementary Materials Table S2).

E6/E7 mRNA HR HPV 16 is an indicator for diagnosing HSIL, and a statistically significant predictive value was determined for all types of cytological findings, NILM, ASCUS, and LSIL. In a patient with confirmed HPV 16 oncogenic transcripts, the probability of diagnosing HSIL is 50-fold higher than the probability of diagnosing NILM. Patients with a positive result of E6/E7 mRNAs HR HPV 16 are 12-fold more likely to be diagnosed with HSIL compared to the detection of ASCUS, while in patients with the same result, the probability of detection of HSIL is 6-fold higher than the probability of detection of LSIL (Supplementary Materials Table S3).

A statistically significant predictive value for the age category was determined in all cytological groups, NILM, ASCUS, and LSIL. The probability of detecting HSIL concerning normal results in HR-HPV-positive patients is 6.3-fold higher if they are ≥ 45 years of age than women under 30. A similar prediction for the detection of HSIL was shown concerning ASCUS (6.5-fold). Patients from the oldest age category have a 3.4-fold higher probability of detecting HSIL concerning LSIL than the youngest (Supplementary Materials Table S4).

The predictive model contains four independent variables: HR HPV DNA 16, E6/E7 mRNA HR HPVs, E6/E7 mRNA HR HPV 16, and age category. The HSIL lesion, as a representative of a high degree of cervical atypia, represented a dependent variable concerning all of the analyzed relevant factors. Multivariate logistic regression analysis was applied to all of the analyzed factors to construct a predictive model and named the most relevant predictors for the development of HSIL (Table 12).

Analyzing the mutual influence of the examined relevant factors for diagnosing HSIL compared to the probability of diagnosing a normal cytological finding, the strongest statistically significant predictor was determined to be the oncogenic activity of the HPV 16 genotype. If it is detected, the probability of diagnosing HSIL concerning the probability of diagnosing normal results increases 19-fold (OR = 19.10; CI (95%): 1.54–236.98; *p* = 0.022). A statistically significant but almost three-fold weaker predictor for the detection of HSIL compared to NILM is the patient belonging to the oldest age category (OR = 6.65; CI (95%): 1.66–26.60; *p* = 0.007), while female patients from the age category 31–44 years have a slightly lower statistically significant probability (OR = 5.38; CI (95%): 1.36–21.30; *p* = 0.016) for the detection of the same lesion. The age category was determined as the strongest statistically significant predictor by analyzing the mutual influence of relevant factors for diagnosing HSIL concerning the probability of detecting ASCUS. HR HPV DNA-positive patients belonging to the oldest age category (≥45 years) have an 8.7-fold higher probability of being diagnosed with HSIL compared to the probability of being diagnosed with ASCUS compared to patients belonging to the younger age category (OR = 8.74; CI (95%): 2.15–35.57; *p* = 0.002). Female patients with the proven oncogenic activity of HPV 16 have a probability of detecting HSIL 6.4-fold higher compared to the probability of being diagnosed with ASCUS (OR = 6.38; CI (95%): 1.22–33.54; *p* = 0.029). By analyzing the mutual influence of relevant factors for diagnosing HSIL concerning the probability of detection of LSIL, the strongest statistically significant predictor was determined to be the oncogenic activity of HPV 16 (OR = 5.10; CI (95%): 1.09–23.83; *p* = 0.038), while weaker statistically significant predictability is shown by the patient’s belonging to a specific age category. Female patients belonging to the oldest age category (≥45 years) have a 3.7-fold greater probability of detecting an HSIL change compared to the detection of LSIL compared to the younger age category (≤30 years) (OR = 3.72; CI (95 %): 1.16–11.92; *p* = 0.027) (Table 12).

## 4. Discussion

Persistent infection caused by HR HPV is the leading risk factor for developing cervical intraepithelial lesions and cervical cancer (reviewed in [20]). Many countries have introduced screening programs based on the cytomorphological examination of cervical samples using the PAP test during the last 60 years to reduce the morbidity and mortality caused by cervical cancer. However, this procedure has shown less than optimal sensitivity (50%) and high inter- and intra-individual variability [21,22]. HPV DNA detection and genotyping provide an efficient screening method and enable risk stratification. However, just proving the presence of the HPV genome in a cervical epithelium does not provide insight into the type of infection. It does not answer whether there is a transient or persistent infection in which the virus is actively multiplying and in which there is a high risk of cancer developing (reviewed in [23]). It was established that cervical carcinogenesis is strongly associated with the HPV-caused infection in which the transcription of E6 and E7 HR HPV oncogenes occurs, with the consequent increase of their mRNA and protein levels. For this reason, the detection of E6 and E7 mRNA of HR HPVs can serve as a promising biomarker of their persistence and oncogenic activity (reviewed in [23]), which could enable a better assessment of the progression to high-grade cervical intraepithelial lesions and, in this regard, significantly influence the algorithm for monitoring patients (reviewed in [18,23,24]).

In our study, 365 female patient samples from Serbia were tested for the twelve HR HPV genotypes. It should be emphasized that the study has limitations. Used HPV tests are non-validated according to European Guidelines for use in primary screening (Meijer HPV test criteria) or according to the VALidation of HPV GENoyping Tests (VALGENT) protocols [25]. The commercial molecular HPV test was chosen for research according to Serbia’s general requirements for molecular diagnostics and the limited research expenses. From the total number of tested samples, 246 (67.4%) samples were positive for at least one of the 12 tested HR genotypes. These results agree with previous studies in the same area and European countries, where a high prevalence of HPV was registered. Studies in Serbia have reported that the presence of HR HPV infections range from 50% to 79% of women [26,27]. In the countries of our region, such as Croatia, a similar prevalence was shown (59%) [28] and in Bulgaria (61%) [29], while a slightly lower prevalence was registered in Italy (53%) [30]. Contrarily, a low prevalence of HPV infection was registered in Western and North European countries. Some of them are Great Britain (20.6%), Sweden (9.7%), and the Netherlands (3.8%) [7].

HPV genotype 16 (38%) emerged as the most frequently detected genotype in the study group, in concordance with previous reports [26,27]. The presence of HPV 16 is more often present (76%) in high-grade lesions compared to lower-grade lesions of the cervical epithelium. Studies on the HPV 16 genotype’s prevalence concerning cytological findings confirm these results (reviewed in [6]). The following frequencies also represented the Alpha-9 genotype, HPV 31 (17%) and HPV 33 (9%). A meta-analysis study on five continents shows that HPV 31 is especially frequent in Europe [31]. The results of this research indicate that the frequency of the HPV 31 genotype statistically significantly decreases with the progression of the intraepithelial lesion. The decreasing trend of the same genotype’s presence according to the degree of severity of the cytological lesion, more precisely, a lower prevalence in cancers compared to precancerous lesions, is observed in research from other studies [32,33,34]. Like HPV 31, a prevalence decrease of HPV 33 in HSIL compared to normal cytology was also registered in this research. Globally, HPV 33 ranks fourth in frequency and is responsible for 4.2% of all registered cervical cancers [6]. The surprising fact is a markedly high prevalence (9%) of the Alpha-5 genotype HPV 51 in our research. In the context of the causative agents of cervical cancer, HPV 51 is not included in the top ten most frequent HPV genotypes registered worldwide [6]. However, the detection of this genotype within this research, as well as previous studies from our region [26] and certain European countries [35,36,37,38,39,40,41], places it among the first four most prevalent genotypes detected in precancerous lesions of the cervix. Since HPV vaccines do not protect against all oncogenic HPVs, such as HPV 51, a complete understanding of its oncogenic activity is particularly significant [42]. The remaining tested genotypes (HR HPV 52, 56, 45, 18, 59, 58, 39, and 35) were present in a low percentage which is in concordance with previous reports [26]. Although HPV 18 is considered to be responsible for 15% of invasive cervical cancers, it is essential to note that its prevalence is similar to some studies from neighboring countries [29,43,44]; we found that in our area, HPV 18 was present at a lower percentage. Its frequency (3.6%) was 10-fold lower than that of HPV 16 (Figure 1). The HPV vaccine was introduced in over half of the WHO member countries in 2020 [5]. There is scientific data from numerous countries that have implemented HPV vaccines in their routine immunization programs on decreasing the burden of cervical HPV infections and precancers [45]. According to our data, using the nine-valent vaccine could prevent more than 80% of the cervical precancerous lesions identified in this study.

The presence of HR HPVs determined further examination of their oncogenic potential. In our study, the expression of E6/E7 mRNA HR HPV was identified in 67% of the HPV-positive samples (Table 6). The percentage of expression of the E6 and E7 HPV-examined HR HPVs is proportional to the degree of severity of the cervical lesion (Table 7). It can be observed that approximately every tenth HPV-infected woman (11%) with normal cytological findings is infected with an oncogenically expressed HPV. At the same time, in HSIL, this relationship is the opposite. Namely, the absence of indicators of HPV oncogenic activity is detected in approximately every tenth woman with HSIL status (11.1%). An undoubted trend in E6/E7 mRNA HR HPV positivity with increasing cytology severity has been observed in the data of studies conducted in different regions of the world [46,47,48,49,50]. In agreement with the report of Argyri et al. (2013) and according to the mRNA test, E6/E7 expression was prevalent in 9.1% of women with normal cytology, similar to our study [51]. However, other previously published data indicated the prevalence of those transcripts in a lower proportion of women with normal cytology findings than our study (0%) [52].

In our study, HPV 16 constituted 29.7%, followed by genotype 31 (19.2%), genotype 33 (9.3%), and genotype 51 (8.7%). The results showed that approximately every second HPV 16 genotype is oncogenically expressed (48.6%). Our data agree with the results reported in other studies. Rossi et al. (2017) observed a positivity rate for E6/E7 mRNA HR HPV, ranging from 58% to 77% in HPV-DNA-positive women [50]. A study by Tüney et al. (2017) in Turkey registered 55.6% E6/E7 mRNA HR HPVs, where HPV genotype 16 constituted 57.8% [24]; similar to that, Bruno et al. (2018) found that the HPV 16 genotype was the most oncogenetically active [46]. As confirmed in this research, it is also stated by several other authors that the transcription product HPV 16 is most often detected, which indicates that the tendency of expression of the essential oncogenes of this genotype is significantly higher compared to the other examined genotypes [24,53,54], which gives it the status of the HPV with the most carcinogenic potential [55]. The oncogenic activity of HPV 31 was detected in approximately every fifth (19.2%) HPV-DNA-positive sample. Contrary to HPV 16, the opposite trend is observed with the degree of cervical lesion severity. It can be assumed that the negative transcriptional status of E6 and E7 oncogenes is more prevalent due to the presence of an episomal form of the virus or an established transcriptional control that enables the spontaneous elimination of infection. Within this research, the results of E6/E7 mRNA HPV 51 detection are approximately the same as those of HPV 33. The distribution of oncogenic activity of these two genotypes is approximately the same across cytological groups and remains at a low level (2.2–11.4%) (Table 7). Other studies have registered different percentage ranges of transcriptional detection of oncogenes of a particular genotype (HPV 33), from its absence [52] to complete expression of 100% [55]. The reason for those differences can be explained by the variations in their number in the total sample [55]. Furthermore, the total oncogenic activity increases with the degree of cervical lesion severity (60–100%). The oncogenic activity of individual genotypes (20–86%) is higher than that of multiple genotypes (0–40%) in the same type of lesion and increases with the degree of its severity (Table 8). These data are supported by the literature. Each of the detected genotypes is considered to have an independent mechanism of action in oncogenesis [56,57,58,59], which supports the hypothesis that different cells being infected with different viral genotypes rather than their intracellular coexistence is possible [60].

To analyze the oncogenic activity of the four most frequently diagnosed HR HPVs related to the age categories, a statistically significantly higher prevalence of positive E6/E7 mRNA HPV 16 findings was observed in the older age groups (Table 9). In agreement with this result, studies have shown that E6 and E7 mRNA HR HPV detection is significantly higher after 30 years [48,61]. The same result is supported by population-based cohort studies, which state that the majority of young women have an asymptomatic HPV infection which, thanks to the immune response, acquires transitory status [49,62].

The growing interest in molecular diagnostics methods has led various authors to compare the characteristics of the HPV DNA test with the mRNA test, evaluating the diagnostic accuracy in identifying high-grade cervical lesions. Our data showed that the specificity (89%) and PPV (70–79%) of the mRNA test are statistically significantly higher, while the same test has a statistically significantly lower sensitivity than the HPV DNA test. These results agree with the statements of different authors, who emphasized slightly lower values of the sensitivity of the mRNA test compared to the sensitivity of the HPV DNA test. At the same time, the specificity is expressed at a higher level [48,49] (reviewed in [63]). In contrast, others suggest that the sensitivity is similar and that its application would significantly improve the assay’s specificity characteristics [49]. Studies evaluating the reliability of the mRNA assay showed heterogeneous findings (reviewed in [32]). The meta-analysis results indicated that the obtained sensitivity value ranged from 41% to 95%, while the registered specificity was 42–97% (reviewed in [64]). The observed difference is explained by the heterogeneous participation of cervical pathology [64] and methodological quality in different studies, which highlights the limitations in the general interpretation of these test characteristics [32]. Although the results are presented broadly, they maintain a unique trend and suggest that the mRNA test over the HPV DNA test improves specificity [64]. By comparing the clinical characteristics of the test, it can be concluded that the detection of E6 and E7 mRNA HR HPV compared to HPV DNA represents a much better marker for more accurate screening of high-grade cellular atypia of the cervix (reviewed in [32]), which make it an appropriate tool for the secondary screening of cervical cancer [47,64].

In our study, the results of the ROC curve analysis indicated that the probability of detecting HPV infection with the mRNA test (81%, AUC = 0.812) in patients with high-grade lesions is higher than the possibility of diagnosing them with the HPV DNA test (74%, AUC = 0.740). The obtained results are in line with the other studies emphasizing the potential usefulness of this test. Sun et al. (2021) also found higher AUC values for the mRNA assay compared to the DNA HPV (0.929 vs. 0.833) [65], while according to Yao et al. (2017), the clinically relevant portion of the AUC of mRNA was 0.721 [66].

According to the previously established degree of influence on diagnosing HSIL, relevant factors were selected in our research. Factors that represent an increased risk for more severe cervical changes were gradually examined (the HPV 16 genotype, the total oncogenic activity of all of the tested HPV genotypes, the HPV 16 oncogenic activity, and the age category of the patient).

Firstly, the HPV-DNA-16-positive results have a statistically significant predictive value for diagnosing HSIL (Table S1). In the same context, the study of Bruno et al. (2018) stated that the presence of the HPV 16 genotype was associated with a five-fold higher risk of developing a high-grade lesion compared to women with the presence of another HPV genotype [46]. Similarly, the data from a recent study indicate that type-specific HPV persistence predicted high-grade lesions, with HPV 16 being the most common type [67].

Secondly, the oncogenic activity of all of the tested HR HPVs has a statistically significant predictive value for diagnosing HSIL (Table S2). The obtained results support the statements related to the research on the importance of oncogenic activity, which show that the detection of E6 and E7 mRNA HR HPV could have a prognostic value in monitoring the development of carcinogenesis [68,69]. The results of the study by Fontecha et al. (2016) [55] showed that in the highest percentage of E6 and E7 mRNA HPV-positive women, the progression of the lesion is diagnosed over time (53%), followed by the persistence of abnormal cytological findings (42%), while regression was recorded in 10-fold lower cases (4%).

Furthermore, in patients with positive results of indicators of the oncogenic activity of HPV 16, a statistically significantly higher probability of diagnosing a high-grade lesion was determined in all types of cytological groups (Table S3). According to the previous insights into the published statements, one of the few prospective follow-up studies that dealt with the predictive value of E6 and E7 mRNA HPV 16 indicated that through the detection of this biomarker, it is possible to identify 87.5% of the HPV infections that progressed. In this case, the risk of progression of negative cytology and low-grade cervical lesions was 10-fold higher than that detected in mRNA-negative women during a follow-up period of 35 months [70]. This is supported by the results of Johansson et al. (2015), which indicated that the absence of E6/E7 mRNA HPV demonstrated a high negative predictive value for the future development of high-grade lesions of the cervix among HR-HPV-DNA-positive women with ASCUS and LSIL [68].

Within the results of this research, a statistically significant final model with all of the predictors was constructed, which shows the strength of the potential of the examined factors, that is, biomarkers for diagnosing precancerous lesions in women with HR HPV infection. Looking at each cytological group individually, two independent variables made a statistically significant contribution to the model and were thus named as the strongest predictors. These are the oncogenic activities of HR HPV 16 and the age category (≥45 years). It is known that the presence of HPV infection is confirmed in all age categories. However, belonging to a particular age group is a determinant significantly associated with the risk of acquiring this infection, depicting the peak in prevalence, which generally takes place around 20–25 years of age. For lesions to progress to more severe forms of cervical disease, a period of 5–14 years is necessary, during which the infection persists and the process of oncogenesis takes place [71]; the results of this research confirm the stated findings. In the examined women, the age category (≥45 years) is a statistically significant prognostic factor for diagnosing HSIL in all of the cytological groups (Table 12 and Table S4). Loopik et al. (2020) [58] indicated that the risk of progression of an existing high-grade lesion increases with age, i.e., in women over 50, the risk of developing cervical cancer increases by seven fold. In addition, the risk of developing cervical abnormalities and the need to use an mRNA test in the diagnostic protocol of HPV-DNA-positive postmenopausal women with normal cytology is emphasized by Asciutto et al. (2020) [72].

## 5. Conclusions

In summary, this study describes the detection rates of the most common HR HPVs (16, 31, 33, and 51) and E6/E7 mRNA HR HPV expression in 365 Serbian women who showed normal and abnormal cytological findings. Those HR HPV genotypes are oncogenically active in more than half of the examined cases, and the detected oncogenic activity has predictive potential in diagnosing high-grade cervical intraepithelial lesions. According to the constructed predictive model, the oncogenic activity of HPV 16 and age are risk factors with the strongest predictive values for developing those lesions. Thus, our data indicate that mRNA testing may be more relevant than HPV DNA for assessing lesion grade and diagnosing and monitoring women at risk of progressive cervical disease. This way, the mRNA test as a tool for better risk stratification of HPV infection could overcome unnecessary examinations, increased costs, and patient anxiety. However, further follow-up studies are needed to determine the clinical utility of the mRNA HR HPV test.

## Figures and Tables

**Table 1:** Primer and probe sequences used for the RT-PCR analysis.

Gene	Primer and Probe Sequences (5′–3′)
E6/E7 HPV 16	F: TTGCAGATCATCAAGAACACGTAGA
	R: CAGTAGAGATCAGTTGTCTCTGGTTGC
	P: FAM-AATCATGCATGGAGATACACCTACATTGCATGA-TAMRA
E6/E7 HPV 31	F: ATTCCACAACATAGGAGGAAGGTG
	R: CACTTGGGTTTCAGTACGAGGTCT
	P: FAM-ACAGGACGTTGCATAGCATGTTGGA-TAMRA
E6/E7 HPV 33	F: ATATTTCGGGTCGTTGGGCA
	R: ACGTCACAGTGCAGTTTCTCTACGT
	P: FAM-GGACCTCCAACACGCCGCACA-TAMRA *
E6/E7 HPV 51	F: AAAGCAAAAATTGGTGGACGA
	R: TGCCAGCAATTAGCGCATT
	P: FAM-CATGAAATAGCGGGACGTTGGACG-TAMRA

F—forward; R—reverse; P—TaqMan probe; *—antisense; FAM—6-carboxyfluorescein; TAMRA—6-carboxytetramethylrhodamine.

**Table 2:** Distribution of the most prevalent HR HPVs (HR HPV 16, 31, 33, and 51) in single and multiple infections.

HPV Infection	HR HPV DNA	*n* (%)	*n* (%)
Single	16	83 (48.3)	145 (84.3)
	31	28 (16.3)	
	33	18 (10.5)	
	51	16 (9.3)	
Multiple	16, 31	13 (7.6)	27 (15.7)
	16, 51	6 (3.5)	
	31, 33	3 (1.7)	
	16, 33	2 (1.2)	
	31, 51	2 (1.2)	
	16, 31, 33	1 (0.6)	
Total:		172 (100)	172 (100)

**Table 3:** Cervical cytology and age of female patients diagnosed with the most prevalent HR HPVs.

Most Prevalent HR-HPV-DNA-Positive Women	*n* (%)
Cytology	
NILM	29 (16.9)
ASCUS	46 (26.7)
LSIL	44 (25.6)
HSIL	53 (30.8)
Total:	172 (100)
Age	
≤30	68 (36.5)
31–44	62 (36.0)
≥45	42 (24.4)
Mean age (years, SD))	36.7 (12.6)

SD—standard deviation; NILM—negative for an intraepithelial lesion or malignancy; ASCUS—atypical squamous cells of unknown significance; LSIL—low-grade squamous intraepithelial lesions; HSIL—high-grade squamous intraepithelial lesions.

**Table 4:** Distribution of the most frequently detected HR HPVs according to cytology.

HR HPV DNA		Cytology				χ^2^	*p*
		NILM	ASCUS	LSIL	HSIL		
		*n* (%)	*n* (%)	*n* (%)	*n* (%)		
HPV 16	+	13 (44.8)	28 (60.9)	24 (54.5)	40 (75.5)	8.628	0.035 *
	−	16 (55.2)	18 (39.1)	20 (45.5)	13 (24.5)		
HPV 31	+	11 (37.9)	16 (34.8)	14 (31.8)	6 (11.3)	10.214	0.017 *
	−	18 (62.1)	30 (65.2)	30 (68.2)	47 (88.7)		
HPV 33	+	6 (20.7)	6 (13.0)	5 (11.4)	7 (13.2)	1.398	0.706
	−	23 (79.3)	40 (87.0)	39 (88.6)	46 (86.8)		
HPV 51	+	4 (13.8)	5 (10,9)	8 (18.2)	7 (13.2)	1.045	0.790
	−	25 (86.2)	41 (89.1)	36 (81.8)	46 (86.8)		
Total:		29 (100)	46 (100)	44 (100)	53 (100)		

* *p* < 0.05. NILM—negative for an intraepithelial lesion or malignancy; ASCUS—atypical squamous cells of unknown significance; LSIL—low-grade squamous intraepithelial lesions; HSIL—high-grade squamous intraepithelial lesions.

**Table 5:** Age-specific distribution of female patients with different cytological groups and genotypes.

HR HPV-Positive Women	Age Group (Years)			Total *n* (%)	χ^2^	*p*	Mean Age (years, (SD))	#	*p*
	≤30	31–44	≥45						
	*n* (%)	*n* (%)	*n* (%)						
Cytology									
NILM	19 (27.9)	5 (8.1)	5 (11.9)	29 (16.9)	29.500	0.000 ***	30.9 (12.2)	9.321	0.000 ***
ASCUS	21 (30.9)	21 (33.9)	4 (9.5)	46 (26.7)			33.4 (9.2)		0.000 ***
LSIL	17 (25.0)	18 (29.0)	9 (21.5)	44 (25.6)			35.9 (11.5)		0.012 *
HSIL	11 (16.2)	18 (29.0)	24 (51.1)	53 (30.8)			43.4 (6.8)		-
Total:	68 (39.5)	62 (36.1)	42 (24.4)	172 (100)					
Genotype								§	
HR HPV 16	41 (50.6)	39 (52.0)	25 (56.8)	105 (52.5)	0.147	0.929	36.9 (12.9)	0.289	0.773
HR HPV 31	24 (29.6)	15 (20.0)	8 (18.2)	47 (23.5)	3.930	0.140	33.1 (10.8)	2.317	0.022 *
HR HPV 33	10 (12.3)	10 (13.3)	4 (9.1)	24 (12.0)	0.963	0.618	34.1 (11.2)	1.077	0.283
HR HPV 51	6 (7.4)	11 (14.7)	7 15.9	24 (12.0)	2.489	0.298	40.5 (12.7)	1.610	0.109
Total:	81 (40.5)	75 (37.5)	44 (22.0)	200 (100)					

SD—Standard Deviation; #—ANOVA; §—t test; * *p* < 0.05; *** *p* < 0.001.

**Table 6:** Analyses of E6/E7 mRNA HPV 16, 31, 33, and 51 in cervical samples.

E6/E7 mRNA HPV Genotypes		Genotypes *n* (%)	HR HPV 16, 31, 33, 51 Cervical Samples (*n* = 291)		E6/E7 mRNA HR HPV/Most Prevalent HR-HPV-DNA-Positive Samples (%)	E6/E7 mRNA HR HPV Positive/HR-HPV-DNA-Positive (%)
			Positive *n* (%)	Negative *n* (%)		
HPV 16	+	51 (25.5)	51 (48.6)	0 (0.0)	29.7 (51/172)	48.5 (51/105)
	−	149 (74.5)	54 (51.4)	186 (63.9)		
	Total:	200 (100)	105 (36.1)	186 (63.9)		
HPV 31	+	33 (16.5)	33 (70.2)	0 (0.0)	19.2 (33/172)	70.2 (33/47)
	−	167 (83.5)	14 (29.8)	244 (83.8)		
	Total:	200 (100)	47 (16.2)	244 (83.8)		
HPV 33	+	16 (8.0)	16 (66.7)	0 (0.0)	9.3 (16/172)	66.7 (16/24)
	−	184 (92.0)	8 (33.3)	267 (91.8)		
	Total:	200 (100)	24 (8.2)	267 (91.8)		
HPV 51	+	15 (7.5)	15 (62.5)	0 (0.0)	8.7 (15/172)	62.5 (15/24)
	−	185 (92.5)	9 (37.5)	267 (91.8)		
	Total:	200 (100)	24 (8.2)	267 (91.8)		
Total E6/E7 mRNA genotypes		+	115 (57.5)	0 (0.0)	66.9 (115/172)	57.5 (115/200)
		-	85 (42.5)	119 (100)		

**Table 7:** Distribution of E6/E7 mRNA HR HPV according to cytology.

E6/E7 mRNA HPV Genotypes		Cytology				Total *n* (%)	χ^2^	*p*
		NILM *n* (%)	ASCUS *n* (%)	LSIL *n* (%)	HSIL *n* (%)			
HPV 16	+	1 (3.4)	6 (13.0)	10 (22.7)	34 (64.2)	51 (29.7)	46.881	0.000 ***
	-	28 (96.6)	40 (87.0)	34 (77.3)	19 (35.8)	121 (70.3)		
	Total:	29 (100)	46 (100)	44 (100)	53 (100)	172 (100)		
HPV 31	+	7 (24.1)	12 (26.1)	10 (22.7)	4 (7.5)	33 (19.2)	6.858	0.077
	-	22 (75.9)	34 (73.9)	34 (77.3)	49 (92.5)	139 (80.8)		
	Total:	29 (100)	46 (100)	44 (100)	53 (100)	172 (100)		
HPV 33	+	3 (10.3)	3 (6.5)	5 (11.4)	5 (9.4)	16 (9.3)	0.682	0.878
	-	26 (89.7)	43 (93.5)	39 (88.6)	48 (90.6)	156 (90.7)		
	Total:	29 (100)	46 (100)	44 (100)	53 (100)	172 (100)		
HPV 51	+	3 (10.3)	1 (2.2)	5 (11.4)	6 (11.3)	15 (8.7)	-	-
	-	26 (89.7)	45 (97.8)	39 (88.6)	47 (88.7)	157 (91.3)		
	Total:	29 (100)	46 (100)	44 (100)	53 (100)	172 (100)		
Cervical samples								
E6/E7 mRNA HPVs	+	13 (10.9)	20 (29.4)	30 (60.0)	48 (88.9)	111 (38.1)		
	-	106 (89.1)	48 (70.6)	20 (40.0)	6 (11.1)	180 (61.9)	108.623	0.000 ***
	Total:	119 (100)	68 (100)	50 (100)	54 (100)	291 (100)		

*** *p* < 0.001. NILM—negative for intraepithelial lesion or malignancy; ASCUS—atypical squamous cells of unknown significance; LSIL—low-grade squamous intraepithelial lesions; HSIL—high-grade squamous intraepithelial lesions.

**Table 8:** Analysis of the oncogenic activity of multiple infections of the most prevalent HR HPV.

Cytology	HR HPV DNA	f1	Multiple E6/E7 mRNA HR HPV	f2	Single E6/E7 mRNA HR HPV	f3	Multiple E6/E7 mRNA HR HPV * (%)	Single E6/E7 mRNA HR HPV ** (%)	Total Oncogenic Activity
NILM	16, 31	1	-	0	-	0	40.0	20.0	60.0
	16, 51	1	-	0	51	1			
	31, 33	2	31, 33	1	-	0			
	31, 51	1	31, 51	1	-	0			
	Total:	5	Total:	2	Total:	1			
ASCUS	16, 31	6	-	0	16	1	22.2	44.4	66.6
					31	3			
	16, 51	1	-	0	-	0			
	31, 33	1	31, 33	1	-	0			
	31, 51	1	31, 51	1	-	0			
	Total:	9	Total:	2	Total:	4			
LSIL	16, 31	3	-	0	16	1	0.0	83.3	83.3
					31	2			
	16, 31, 33	1	-	0	33	1			
	16, 51	2	-	0	51	1			
	Total:	6	Total:	0	Total:	5			
HSIL	16, 31	3	16, 31	1	16	1	14.3	85.7	100
					31	1			
	16, 33	2	-	0	16	1			
					33	1			
	16, 51	2	-	0	16	1			
					51	1			
	Total:	7	Total:	1	Total:	6			

* (f2/f1) × 100; ** (f3/f1) × 100; NILM—negative for an intraepithelial lesion or malignancy; ASCUS—atypical squamous cells of unknown significance; LSIL—low-grade squamous intraepithelial lesions; HSIL—high-grade squamous intraepithelial lesions.

**Table 9:** Analyses of E6/E7 mRNA HPVs according to age.

E6/E7 mRNA HR HPV		Age (years)			Total	χ^2^	*p*
		≤30	31–44	≥45			
		*n* (%)	*n* (%)	*n* (%)	*n* (%)		
HPV 16	+	13 (19.1)	20 (32.3)	18 (42.9)	51 (29.7)	7.331	0.026 *
	-	55 (80.9)	42 (67.7)	24 (57.1)	121 (70.3)		
	Total:	68 (100)	62 (100)	42 (100)	172 (100)		
HPV 31	+	16 (23.5)	10 (16.1)	7 (16.7)	33 (19.2)	1.373	0.503
	–	52 (76.5)	52 (83.9)	35 (83.3)	139 (80.8)		
	Total:	68 (100)	62 (100)	42 (100)	172 (100)		
HPV 33	+	7 (10.3)	6 (9.7)	3 (7.1)	16 (9.3)	0.322	0.851
	–	61 (89.7)	56 (90.3)	39 (92.9)	156 (90.7)		
	Total:	68 (100)	62 (100)	42 (100)	172 (100)		
HPV 51	+	3 (4.4)	5 (8.1)	7 (16.7)	15 (8.7)	4.951	0.084
	–	65 (95.6)	57 (91.9)	35 (83.3)	157 (91.3)		
	Total:	68 (100)	62 (100)	42 (100)	172 (100)		

* *p <* 0.05.

**Table 10:** Clinical characteristics of HR HPV DNA and E6/E7 mRNA HPV tests.

Test	Cytology	Sensitivity	CI	Specificity	CI	PPV	CI	NPV	CI
		(%)	(95%)	(%)	(95%)	(%)	(95%)	(%)	(95%)
HPV DNA	ASCUS	67.6 ***	55.2–78.5	75.6	66.9–83.0	61.3	49.4–72.4	80.4 *	71.8–87.3
	LSIL	88.0 **	75.7–95.5	75.6	66.9–83.0	60.3	48.1–71.6	93.8 *	86.9–97.7
	HSIL	98.2	90.1–100	75.6	66.9–83.0	64.6	53.3–74.9	98.9	94.0–100
E6/E7 mRNA HPV	ASCUS	29.4	19.0–41.7	89.1 **	82.0–94.0	60.6	42.1–77.1	68.8	60.9–76.0
	LSIL	60.0	45.2–73.6	89.1 **	82.0–94.0	69.8 ***	53.9–82.8	84.1	76.6–90.0
	HSIL	88.9	77.4–95.8	89.1 **	82.0–94.0	78.7 ***	66.3–88.1	94.6	88.7–98.0

* *p* < 0.05; ** *p* < 0.005; *** *p* < 0.001. CI (95%)—95% confidence interval; PPV—positive predictive value; NPV—negative predictive value; NILM—negative for intraepithelial lesion or malignancy; ASCUS—atypical squamous cells of unknown significance; LSIL—low-grade squamous intraepithelial lesions; HSIL—high-grade squamous intraepithelial lesions.

**Table 11:** Performance of E6/E7 mRNA HR HPV and HR HPV DNA tests in HSIL.

HSIL	AUC ± SE	*p*	CI (95%)
E6/E7 mRNA HR HPV	0.812 ± 0.031	0.000 ***	0.752–0.871
HR HPV DNA	0.740 ± 0.030	0.000 ***	0.680–0.799

AUC—area under the ROC curve; SE—standard error; *** *p* < 0.001; CI (95%)—95% confidence intervals.

**Table 12:** Analysis of the mutual influence of relevant factors for HSIL development.

HSIL			OR	CI (95%)	*p*
NILM	HR HPV DNA 16	+	1.627	0.351–7.531	0.534
		–	1.00 ^a^		
	E6/E7 mRNA HR HPV	+	3.989	0.843–18.882	0.081
		–	1.00 ^a^		
	E6/E7 mRNA HR HPV 16	+	19.099	1.539–236.983	0.022 *
		–	1.00 ^a^		
	Age (years)	≤30	1.00^a^		
		31–44	5.382	1.360–21.296	0.016 *
		≥45	6.654	1.665–26.598	0.007 **
ASCUS	HR HPV DNA 16	+	0.957	0.230–3.988	0.952
		–	1.00 ^a^		
	E6/E7 mRNA HR HPV	+	3.910	0.906–16.871	0.068
		–	1.00 ^a^		
	E6/E7 mRNA HR HPV 16	+	6.384	1.215–33.545	0.029 *
		–	1.00 ^a^		
	Age (years)	≤30	1.00 ^a^		
		31–44	1.401	0.469–4.182	0.546
		≥45	8.738	2.147–35.568	0.002 **
LSIL	HR HPV DNA 16	+	1.009	0.243–4.192	0.990
		–	1.00 ^a^		
	E6/E7 mRNA HR HPV	+	1.636	0.377–7.102	0.511
		–	1.00 ^a^		
	E6/E7 mRNA HR HPV 16	+	5.099	1.091–23.832	0.038 *
		–	1.00 ^a^		
	Age (years)	≤30	1.00 ^a^		
		31–44	1.362	0.464–3.992	0.574
		≥45	3.719	1.161–11.920	0.027 *

OR—Odds ratio; ^a^—reference; CI (95%)—95% confidence interval; * *p* < 0.05; ** *p* < 0.01; NILM–negative for intraepithelial lesion or malignancy; ASCUS–atypical squamous cells of unknown significance; LSIL–low-grade squamous intraepithelial lesions; HSIL–high-grade squamous intraepithelial lesions.

## Data Availability

The data that support the findings of this study are available from the corresponding author upon reasonable request.

## Supplementary Materials

The following supporting information can be downloaded at: https://www.mdpi.com/article/10.3390/diagnostics13050917/s1. Figure S1: Dispersion of Ct values of E6/E7 mRNA HR HPVs. Table S1: Analysis of HR HPV DNA 16’s influence on the diagnosis of HSIL; Table S2: Analysis of total E6/E7 mRNA HR HPV’s influence on the diagnosis of HSIL; Table S3: Analysis of E6/E7 mRNA HR HPV 16’s influence on the diagnosis of HSIL; Table S4: Analysis of the influence of age on the diagnosis of HSIL.

## References

[1] Araldi R.P., Muro S., Assaf R., De Carvalho R.F., Caldas M.A., de Carvalho R., de Souza J.M., Magnelli R.F., Grando D., Roperto F.P. (2017). Papillomaviruses: A Systematic Review. Genet. Mol. Biol..

[2] Gheit T. (2019). Mucosal and Cutaneous Human Papillomavirus Infections and Cancer Biology. Front. Oncol..

[3] Mehta K., Lamins L., Wu T.-C., Chang M.-H., Jeang K.-T. (2021). High-Risk Human Papillomaviruses and DNA Repair. Viruses and Human Cancer: From Basic Science to Clinical Prevention (Recent Results in Cancer Research).

[4] Sung H., Ferlay J., Siegel R.L., Laversanne M., Soerjomataram I., Jemal A., Bray F. (2021). Global Cancer Statistics 2020: GLOBOCAN Estimates of Incidence and Mortality Worldwide for 36 Cancers in 185 Countries. CA. Cancer J. Clin..

[5] Rancic N.K., Miljkovic P.M., Deljanin Z.M., Marinkov-Zivkovic E.M., Stamenkovic B.N., Bojanovic M.R., Jovanovic M.M., Miljkovic D.P., Stankovic S.M., Otasevic S.A. (2022). Knowledge about HPV Infection and the HPV Vaccine among Parents in Southeastern Serbia. Medicina.

[6] Bruni L., Albero G., Serrano B., Mena M., Collado J., Gómez D., Muñoz J., Bosch F., de Sanjosé S. Human Papillomavirus and Related Diseases in Serbia. https://hpvcentre.net/statistics/reports/SRB.pdf.

[7] Bruni L., Albero G., Serrano B., Mena M., Collado J., Gómez D., Muñoz J., Bosch F., de Sanjosé S. Human Papillomavirus and Related Diseases in the World. https://hpvcentre.net/statistics/reports/XWX.pdf.

[8] Rosenblum H.G., Lewis R.M., Gargano J.W., Querec T.D., Unger E.R., Markowitz L.E. (2021). Declines in Prevalence of Human Papillomavirus Vaccine-Type Infection Among Females after Introduction of Vaccine—United States, 2003–2018. MMWR Surveill. Summ..

[9] Lee L.Y., Garland S.M. (2017). Human Papillomavirus Vaccination: The Population Impact. F1000Research.

[10] Bouvard V., Baan R., Straif K., Grosse Y., Secretan B., El Ghissassi F., Benbrahim-Tallaa L., Guha N., Freeman C., Galichet L. (2009). A Review of Human Carcinogens--Part B: Biological Agents. Lancet Oncol..

[11] Egawa N., Doorbar J. (2017). The Low-Risk Papillomaviruses. Virus Res..

[12] Wang S.S., Hildesheim A. (2003). Chapter 5: Viral and Host Factors in Human Papillomavirus Persistence and Progression. J. Natl. Cancer Inst. Monogr..

[13] McBride A.A. (2017). Mechanisms and Strategies of Papillomavirus Replication. Biol. Chem..

[14] Williams V.M., Filippova M., Soto U., Duerksen-Hughes P.J. (2011). HPV-DNA Integration and Carcinogenesis: Putative Roles for Inflammation and Oxidative Stress. Future Virol..

[15] Fernandes J.V., de Medeiros Fernandes T.A.A., Broeck D.D., Vanden (2012). Human Papillomavirus: Biology and Pathogenesis. Human Papillomavirus and Related Diseases—From Bench to Bedside—A Clinical Perspective.

[16] Münger K., Howley P.M. (2002). Human Papillomavirus Immortalization and Transformation Functions. Virus Res..

[17] Wentzensen N., Arbyn M., Berkhof J., Bower M., Canfell K., Einstein M., Farley C., Monsonego J., Franceschi S. (2017). Eurogin 2016 Roadmap: How HPV Knowledge Is Changing Screening Practice. Int. J. Cancer.

[18] Burger E.A., Kornør H., Klemp M., Lauvrak V., Kristiansen I.S. (2011). HPV MRNA Tests for the Detection of Cervical Intraepithelial Neoplasia: A Systematic Review. Gynecol. Oncol..

[19] Lindh M., Görander S., Andersson E., Horal P., Mattsby-Balzer I., Ryd W. (2007). Real-Time Taqman PCR Targeting 14 Human Papilloma Virus Types. J. Clin. Virol..

[20] Moscicki A.B., Ma Y., Wibbelsman C., Darragh T.M., Powers A., Farhat S., Shiboski S. (2010). Rate of and Risks for Regression of Cervical Intraepithelial Neoplasia 2 in Adolescents and Young Women. Obstet. Gynecol..

[21] Boulet G.A.V., Horvath C.A.J., Berghmans S., Bogers J. (2008). Human Papillomavirus in Cervical Cancer Screening: Important Role as Biomarker. Cancer Epidemiol. Biomarkers Prev..

[22] Wright T.C. (2007). Cervical Cancer Screening in the 21st Century: Is It Time to Retire the Pap Smear?. Clin. Obstet. Gynecol..

[23] Derbie A., Mekonnen D., Woldeamanuel Y., Van Ostade X., Abebe T. (2020). HPV E6/E7 MRNA Test for the Detection of High Grade Cervical Intraepithelial Neoplasia (CIN2+): A Systematic Review. Infect. Agent. Cancer.

[24] Tüney İ., Altay A., Ergünay K., Önder S.Ç., Usubütün A., Salman M.C., Bozdayi G., Karabulut E., Badur O.S., Yüce K. (2017). Hpv Types and E6/E7 MRNA Expression in Cervical Samples from Turkish Women with Abnormal Cytology in Ankara, Turkey. Turkish J. Med. Sci..

[25] Poljak M., Oštrbenk Valenčak A., Gimpelj Domjanič G., Xu L., Arbyn M. (2020). Commercially Available Molecular Tests for Human Papillomaviruses: A Global Overview. Clin. Microbiol. Infect..

[26] Kovacevic G., Nikolic N., Jovanovic-Galovic A., Hrnjakovic-Cvjetkovic I., Vuleta D., Patic A., Radovanov J., Milosevic V. (2016). Frequency of Twelve Carcinogenic Human Papilloma Virus Types among Women from the South Backa Region, Vojvodina, Serbia. Turkish J. Med. Sci..

[27] Kovacevic G., Milosevic V., Nikolic N., Patic A., Dopudj N., Radovanov J., Cvjetkovic I.H., Petrovic V., Petrovic M. (2021). The Prevalence of 30 HPV Genotypes Detected by EUROArray HPV in Cervical Samples among Unvaccinated Women from Vojvodina Province, Serbia. PLoS ONE.

[28] Milutin-Gašperov N., Sabol I., Halec G., Matovina M., Grce M. (2007). Retrospective Study of the Prevalence of High-Risk Human Papillomaviruses among Croatian Women. Coll. Antropol..

[29] Grozdanov P., Zlatkov V., Ganchev G., Karagiosov I., Toncheva D., Galabov A.S. (2014). HPV Prevalence and Type Distribution in Women with Normal or Abnormal Pap Smear in Bulgaria. J. Med. Virol..

[30] Schettino M.T., De Franciscis P., Schiattarella A., La Manna V., Della Gala A., Caprio F., Tammaro C., Ammaturo F.P., Guler T., Yenigün E.H. (2019). Prevalence of HPV Genotypes in South Europe: Comparisons between an Italian and a Turkish Unvaccinated Population. J. Environ. Public Health.

[31] Bruni L., Diaz M., Castellsagué X., Ferrer E., Bosch F.X., De Sanjosé S. (2010). Cervical Human Papillomavirus Prevalence in 5 Continents: Meta-Analysis of 1 Million Women with Normal Cytological Findings. J. Infect. Dis..

[32] Sabol I., Gašperov N.M., Matovina M., Božinovic K., Grubišic G., Fistonic I., Belci D., Alemany L., Džebro S., Dominis M. (2017). Cervical HPV Type-Specific Pre-Vaccination Prevalence and Age Distribution in Croatia. PLoS ONE.

[33] Guan P., Howell-Jones R., Li N., Bruni L., De Sanjosé S., Franceschi S., Clifford G.M. (2012). Human Papillomavirus Types in 115,789 HPV-Positive Women: A Meta-Analysis from Cervical Infection to Cancer. Int. J. Cancer.

[34] Karadža M., Lepej S.Ž., Planinić A., Grgić I., Ćorušić A., Planinić P., Ćorić M., Hošnjak L., Komloš K.F., Poljak M. (2021). Distribution of Human Papillomavirus Genotypes in Women with High-Grade Cervical Intraepithelial Lesions and Cervical Carcinoma and Analysis of Human Papillomavirus-16 Genomic Variants. Croat. Med. J..

[35] Bowden S.J., Fiander A.N., Hibbitts S. (2021). HPV 51: A Candidate for Type-Replacement Following Vaccination?. medRxiv.

[36] Schmitt M., Depuydt C., Benoy I., Bogers J., Antoine J., Arbyn M., Pawlita M. (2013). Prevalence and Viral Load of 51 Genital Human Papillomavirus Types and Three Subtypes. Int. J. Cancer.

[37] Yuce K., Pinar A., Salman M.C., Alp A., Sayal B., Dogan S., Hascelik G. (2012). Detection and Genotyping of Cervical HPV with Simultaneous Cervical Cytology in Turkish Women: A Hospital-Based Study. Arch. Gynecol. Obstet..

[38] Mollers M., Boot Hein J., Vriend Henrike J., King Audrey J., van den Broek Ingrid V.F., van Bergen Jan E.A.M., Brink Antoinette A.T.P., Wolffs Petra F.G., Hoebe Christian J.P.A., Meijer Chris J.L.M. (2013). Prevalence, Incidence and Persistence of Genital HPV Infections in a Large Cohort of Sexually Active Young Women in the Netherlands. Vaccine.

[39] Piana A., Sotgiu G., Cocuzza C., Musumeci R., Marras V., Pischedda S., Deidda S., Muresu E., Castiglia P. (2013). High HPV-51 Prevalence in Invasive Cervical Cancers: Results of a Pre-Immunization Survey in North Sardinia, Italy. PLoS ONE.

[40] Dalgo Aguilar P., Loján González C., Córdova Rodríguez A., Acurio Paéz K., Arévalo A.P., Bobokova J. (2017). Prevalence of High-Risk Genotypes of Human Papillomavirus: Women Diagnosed with Premalignant and Malignant Pap Smear Tests in Southern Ecuador. Infect. Dis. Obstet. Gynecol..

[41] Tang S., Liao Y., Hu Y., Shen H., Wan Y., Wu Y. (2021). HPV Prevalence and Genotype Distribution Among Women From Hengyang District of Hunan Province, China. Front. Public Heal..

[42] Sladič M., Taneska P., Cvjetičanin B., Velikonja M., Smrkolj V., Smrkolj Š. (2022). Cervical Intraepithelial Neoplasia Grade 3 in a HPV-Vaccinated Patient: A Case Report. Medicina.

[43] Učakar V., Poljak M., Klavs I. (2012). Pre-Vaccination Prevalence and Distribution of High-Risk Human Papillomavirus (HPV) Types in Slovenian Women: A Cervical Cancer Screening Based Study. Vaccine.

[44] Ursu R., Onofriescu M., Nemescu D., Iancu L.S. (2011). HPV Prevalence and Type Distribution in Women with or without Cervical Lesions in the Northeast Region of Romania. Virol. J..

[45] Oliveira C.R., Niccolai L.M. (2021). Monitoring HPV Vaccine Impact on Cervical Disease: Status and Future Directions for the Era of Cervical Cancer Elimination. Prev. Med..

[46] Bruno M.T., Ferrara M., Fava V., Rapisarda A., Coco A. (2018). HPV Genotype Determination and E6/E7 MRNA Detection for Management of HPV Positive Women. Virol. J..

[47] Pan D., Zhang C.Q., Liang Q.L., Hong X.C. (2019). An Efficient Method That Combines the ThinPrep Cytologic Test with E6/E7 MRNA Testing for Cervical Cancer Screening. Cancer Manag. Res..

[48] Pruski D., Millert-Kalinska S., Lewek A., Kedzia W. (2019). Sensitivity and Specificity of HR HPV E6/E7 MRNA Test in Detecting Cervical Squamous Intraepithelial Lesion and Cervical Cancer. Ginekol. Pol..

[49] Wang J., Xu X. (2021). The Diagnostic Value of HPV E6/E7 MRNA Test in Young Women with Cervical Squamous Intraepithelial Lesion: A Retrospective Analysis. Research Square. Res. Sq..

[50] Rossi P.G., Bisanzi S., Allia E., Mongia A., Carozzi F., Gillio-Tos A., De Marco L., Ronco G., Gustinucci D., Del Mistro A. (2017). Determinants of Viral Oncogene E6-E7 MRNA Overexpression in a Population- Based Large Sample of Women Infected by High-Risk Human Papillomavirus Types. J. Clin. Microbiol..

[51] Argyri E., Tsimplaki E., Daskalopoulou D., Stravopodis D.J., Kouikoglou O., Terzakis E., Panotopoulou E. (2013). E6/E7 MRNA Expression of High-Risk HPV Types in 849 Greek Women. Anticancer Res..

[52] Baron C., Henry M., Tamalet C., Villeret J., Richet H., Carcopino X. (2015). Relationship Between HPV 16, 18, 31, 33, 45 DNA Detection and Quantitation and E6/E7 MRNA Detection Among a Series of Cervical Specimens With Various Degrees of Histological Lesions. J. Med. Virol..

[53] Dabeski D., Duvlis S., Basheska N., Antovska V., Stojovski M., Trajanova M., Dimitrov G., Dabeski A., Gureva-Gjorgievska N. (2019). Comparison Between HPV DNA Testing and HPV E6/E7 MRNA Testing in Women with Squamous Cell Abnormalities of the Uterine Cervix. Prilozi.

[54] Salimović-Bešić I., Tomić-Čiča A., Smailji A., Hukić M. (2013). Comparison of the Detection of HPV-16, 18, 31, 33, and 45 by Type-Specific DNA- and E6/E7 MRNA-Based Assays of HPV DNA Positive Women with Abnormal Pap Smears. J. Virol. Methods.

[55] Fontecha N., Basaras M., Hernáez S., Andía D., Cisterna R. (2016). Assessment of Human Papillomavirus E6/E7 Oncogene Expression as Cervical Disease Biomarker. BMC Cancer.

[56] Quint W., Jenkins D., Molijn A., Struijk L., Van De Sandt M., Doorbar J., Mols J., Van Hoof C., Hardt K., Struyf F. (2012). One Virus, One Lesion—Individual Components of CIN Lesions Contain a Specific HPV Type. J. Pathol..

[57] van den Heuvel C.N.A.M., Loopik D.L., Ebisch R.M.F., Elmelik D., Andralojc K.M., Huynen M., Bulten J., Bekkers R.L.M., Massuger L.F.A.G., Melchers W.J.G. (2020). RNA-Based High-Risk HPV Genotyping and Identification of High-Risk HPV Transcriptional Activity in Cervical Tissues. Mod. Pathol..

[58] Loopik D.L., IntHout J., Ebisch R.M.F., Melchers W.J.G., Massuger L.F.A.G., Siebers A.G., Bekkers R.L.M. (2020). The Risk of Cervical Cancer after Cervical Intraepithelial Neoplasia Grade 3: A Population-Based Cohort Study with 80,442 Women. Gynecol. Oncol..

[59] Bruno M.T., Scalia G., Cassaro N., Boemi S. (2020). Multiple HPV 16 Infection with Two Strains: A Possible Marker of Neoplastic Progression. BMC Cancer.

[60] Soto-De Leon S., Camargo M., Sanchez R., Munoz M., Perez-Prados A., Purroy A., Patarroyo M.E., Patarroyo M.A. (2011). Distribution Patterns of Infection with Multiple Types of Human Papillomaviruses and Their Association with Risk Factors. PLoS ONE.

[61] Wang H.Y., Lee D., Park S., Kim G., Kim S., Han L., Yubo R., Li Y., Park K.H., Lee H. (2015). Diagnostic Performance of HPV E6/E7 MRNA and HPV DNA Assays for the Detection and Screening of Oncogenic Human Papillomavirus Infection among Woman with Cervical Lesions in China. Asian Pacific J. Cancer Prev..

[62] Mittal S., Basu P., Muwonge R., Banerjee D., Ghosh I., Sengupta M.M., Das P., Dey P., Mandal R., Panda C. (2017). Risk of High-Grade Precancerous Lesions and Invasive Cancers in High-Risk HPV-Positive Women with Normal Cervix or CIN 1 at Baseline—A Population-Based Cohort Study. Int. J. Cancer.

[63] Zorzi M., Del Mistro A., Giorgi Rossi P., Laurino L., Battagello J., Lorio M., Soldà M., Martinotti Gabellotti E., Maran M., Dal Cin A. (2020). Risk of CIN2 or More Severe Lesions after Negative HPV-MRNA E6/E7 Overexpression Assay and after Negative HPV-DNA Test: Concurrent Cohorts with a 5-Year Follow-Up. Int. J. Cancer.

[64] Macedo A.C.L., Gonçalves J.C.N., Bavaresco D.V., Grande A.J., Chiaramonte Silva N., Rosa M.I. (2019). Accuracy of MRNA HPV Tests for Triage of Precursor Lesions and Cervical Cancer: A Systematic Review and Meta-Analysis. J. Oncol..

[65] Sun J., Yue Y., Li R., Sun Q., Hu C., Ge X., Guan Q. (2021). Detection of HPV E6/E7 MRNA in the Diagnosis of Cervical Cancer and Precancerous Lesions after Kidney Transplantation. Am. J. Transl. Res..

[66] Yao Y.L., Tian Q.F., Cheng B., Cheng Y.F., Ye J., Lu W.G. (2017). Human Papillomavirus (HPV) E6/E7 MRNA Detection in Cervical Exfoliated Cells: A Potential Triage for HPV-Positive Women. J. Zhejiang Univ. Sci. B.

[67] Sahlgren H., Elfström K.M., Lamin H., Carlsten-Thor A., Eklund C., Dillner J., Elfgren K. (2020). Colposcopic and Histopathologic Evaluation of Women with HPV Persistence Exiting an Organized Screening Program. Am. J. Obstet. Gynecol..

[68] Johansson H., Bjelkenkrantz K., Darlin L., Dilllner J., Forslund O. (2015). Presence of High-Risk HPV MRNA in Relation to Future High-Grade Lesions among High-Risk HPV DNA Positive Women with Minor Cytological Abnormalities. PLoS ONE.

[69] Liu S., Minaguchi T., Lachkar B., Zhang S., Xu C., Tenjimbayashi Y., Shikama A., Tasaka N., Akiyama A., Sakurai M. (2018). Separate Analysis of Human Papillomavirus E6 and E7 Messenger RNAs to Predict Cervical Neoplasia Progression. PLoS ONE.

[70] Martí C., Marimón L., Glickman A., Henere C., Saco A., Rakislova N., Torné A., Ordi J., Del Pino M. (2021). Usefulness of E7 Mrna in Hpv16-Positive Women to Predict the Risk of Progression to Hsil/Cin2+. Diagnostics.

[71] de Sanjosé S., Brotons M., Pavón M.A. (2018). The Natural History of Human Papillomavirus Infection. Best Pract. Res. Clin. Obstet. Gynaecol..

[72] Asciutto K.C., Borgfeldt C., Forslund O. (2020). 14-Type HPV MRNA Test in Triage of HPV DNA-Positive Postmenopausal Women with Normal Cytology. BMC Cancer.

