# Supplementary material for: E6/E7 mRNA Expression of the Most Prevalent High-Risk HPV Genotypes in Cervical Samples from Serbian Women
Source: Diagnostics (Basel). 2023 Feb 28;13(5):917. doi: 10.3390/diagnostics13050917 (PMC10000477; doi:10.3390/diagnostics13050917)
Supplement: Supplementary file 1 [file diagnostics-13-00917-s001.zip › diagnostics-2135176-supplementary.pdf]

Supplementary Material

E6/E7 mRNA Expression of the Most Prevalent High-Risk HPV Genotypes in Cervical Samples from Serbian Women

Natasa Nikolic[1*], Branka Basica[1], Aljosa Mandic[2,3], Nela Surla[1], Vera Gusman[1,4], Deana Medic[1,4], Tamas Petrovic[5], Mirjana Strbac[1], Vladimir Petrovic[1,6]

1 Institute of Public Health of Vojvodina, 21000 Novi Sad, Serbia
2 Clinic for Oncological Surgery, Oncology Institute of Vojvodina, 21208 Sremska Kamenica, Serbia
3 Department of Gynaecology and Obstetrics, Faculty of Medicine, University of Novi Sad, 21000 Novi Sad, Serbia
4 Department of Microbiology with Parasitology and Immunology, Faculty of Medicine, University of Novi Sad, 21000 Novi Sad, Serbia
5 Scientific Veterinary Institute Novi Sad, 21000 Novi Sad, Serbia
6 Department of Epidemiology, Faculty of Medicine, University of Novi Sad, 21000 Novi Sad, Serbia
* Correspondence: natasa.nikolic@izjzv.org.rs

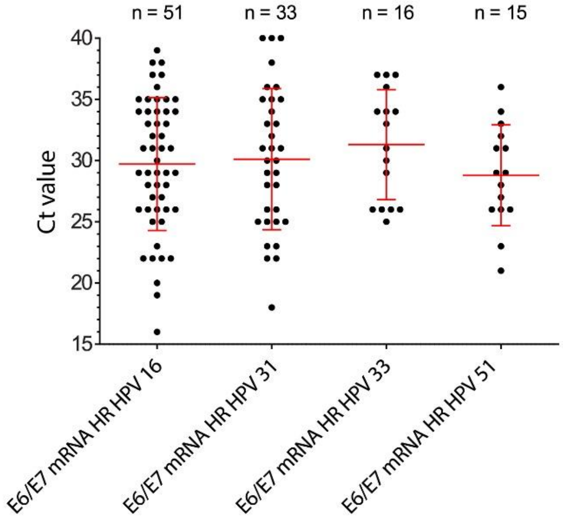

Figure S1. Dispersion of Ct values of E6/E7 mRNA HR HPVs.

Table S1. Analysis of the HR HPV DNA 16 influence on the diagnosis of HSIL.

HSIL			OR	CI (95%)	*p*
NILM	HR HPV DNA 16	+	3.788	1.445–9.919	0.007**
		–	1.00[a]		
ASCUS	HR HPV DNA 16	+	1.976	0.826–4.673	0.121
		–	1.00[a]		
LSIL	HR HPV DNA 16	+	2.564	1.082–6.061	0.032*
		–	1.00[a]		

OR—Odds ratio; [a]—reference; CI (95%)—95% confidence interval; $^{*}p < 0.05$; $^{**}p < 0.01$; NILM—negative for intraepithelial lesion or malignancy; ASCUS—atypical squamous cells of unknown significance; LSIL—low-grade squamous intraepithelial lesions; HSIL—high-grade squamous intraepithelial lesions.

Table S2. Analysis of total E6/E7 mRNA HR HPV influence on the diagnosis of HSIL.

HSIL			OR	CI (95%)	*p*
NILM	E6/E7 mRNA HR HPV	+	6.667	23.255–181.927	0.000***
		–	1.00[a]		
ASCUS	E6/E7 mRNA HR HPV	+	19.231	7.092–52.631	0.000***
		–	1.00[a]		
LSIL	E6/E7 mRNA HR HPV	+	5.319	1.294–14.706	0.001**
		–	1.00[a]		

OR—Odds ratio; [a]—reference; CI (95%)—95% confidence interval; $^{**}p < 0.01$; $^{***}p < 0.001$; NILM—negative for intraepithelial lesion or malignancy; ASCUS—atypical squamous cells of unknown significance; LSIL—low-grade squamous intraepithelial lesions; HSIL—high-grade squamous intraepithelial lesions.

Table S3. Analysis of the E6/E7 mRNA HR HPV 16 influence on the diagnosis of HSIL.

HSIL			OR	CI (95%)	p
NILM	E6/E7 mRNA HR HPV 16	+	50.000	6.289–333.333	0.000***
		−	1.00[a]		
ASCUS	E6/E7 mRNA HR HPV 16	+	11.905	4.273–33.333	0.000***
		−	1.00[a]		
LSIL	E6/E7 mRNA HR HPV 16	+	6.097	2.469–14.925	0.000***
		−	1.00[a]		

OR—Odds ratio; [a]—reference; CI (95%)—95% confidence interval; $^{***}p < 0.001$; NILM—negative for intraepithelial lesion or malignancy; ASCUS—atypical squamous cells of unknown significance; LSIL—low-grade squamous intraepithelial lesions; HSIL—high-grade squamous intraepithelial lesions.

Table S4. Analysis of the age influence on the diagnosis of HSIL.

HSIL			OR	CI (95%)	p
NILM	Age (years)	≤ 30	1.00[a]		
		31–44	1.470	0.582–3.717	0.415
		≥ 45	6.289	2.141–18.518	0.001**
ASCUS	Age (years)	≤ 30	1.00[a]		
		31–44	1.169	0.463–2.958	0.740
		≥ 45	6.536	2.150–20.000	0.001**
LSIL	Age (years)	≤ 30	1.00[a]		
		31–44	2.571	0.330–4.167	0.369
		≥ 45	3.367	1.190–9.524	0.022*

OR—Odds ratio; [a]—reference; CI (95%)—95% confidence interval; $^{*}p < 0.05$; $^{**}p < 0.01$; NILM—negative for intraepithelial lesion or malignancy; ASCUS—atypical squamous cells of unknown significance; LSIL—low-grade squamous intraepithelial lesions; HSIL—high-grade squamous intraepithelial lesions.
